# Preference for community health services in people with chronic diseases: a discrete choice experiment in China

**DOI:** 10.3389/fpubh.2024.1479237

**Published:** 2024-11-20

**Authors:** Ke Wang, Qian Yang, Lei Wan, Jingjing An

**Affiliations:** ^1^Operating Room, Department of Anesthesiology, West China Hospital, Sichuan University/West China School of Nursing, Sichuan University, Chengdu, Sichuan, China; ^2^West China School of Nursing, Sichuan University, Chengdu, China

**Keywords:** chronic diseases, community health services, discrete choice experiment, preferences, China

## Abstract

**Background:**

Primary healthcare policies are widely implemented globally. However, many people with chronic diseases find that community-based chronic disease services do not meet their needs. There is a critical need for more evidence on the sustainability and optimization of chronic disease management in Chinese communities, especially from the demand side. Policymakers require detailed data on the needs of chronic disease patients regarding community health services.

**Methods:**

A discrete choice experiment was conducted to measure the preferences of people with chronic diseases. Researchers recruited participants in Sichuan Province, China, and conducted face-to-face surveys. The mixed logit model evaluated participants’ preferences for six attributes, estimating willingness to pay and relative importance, and performing subgroup analysis based on the initial model results.

**Results:**

A total of 395 respondents participated in this study. Six attributes included all influenced the preference of people with chronic diseases for community health services. The most valued attribute for people with chronic diseases was drug accessibility (coefficient = 2.761, *p* < 0.001), followed by appointment referral (coefficient = 2.385, *p* < 0.001) and traditional Chinese medicine services (coefficient = 1.465, *p* < 0.001). The results were also borne out by the relative importance of attributes. Meanwhile, people with different types of chronic diseases were also most concerned about drug accessibility. There are differences in the willingness to pay for drug accessibility. Type II respondents had a higher WTP for services with high medicine accessibility (92.93 CNY) compared to Type I (67.05 CNY) and Type III (87.70 CNY) respondents.

**Conclusion:**

This study results highlight the importance of drug accessibility, appointment referral services, and traditional Chinese medicine services in community chronic disease management. These findings provide valuable insights for policymakers to optimize the current management of chronic diseases in Chinese communities.

## 1 Introduction

Chronic diseases, characterized by high morbidity, mortality, low awareness, low control rates, and heavy economic burdens, have become a significant public health issue in China and a major health threat worldwide (1, 2). In China, more than 300 million people have been diagnosed with chronic diseases, with a prevalence rate of 34.29% (3, 4). Deaths caused by chronic diseases account for 88.5% of the total mortality (5, 6). By 2026, the prevalence rates of various chronic diseases are projected to increase, with hypertension reaching 27.8% and diabetes rising to 14.4% (2, 7). Moreover, due to imbalanced economic development, irrational resource allocation, and low public health awareness, these trends are worsening, presenting a severe challenge for chronic disease prevention and control in China.

The situation is even more alarming among the older adult due to the aging population. Currently, approximately 180 million older adult individuals in China suffer from chronic diseases, with over two-thirds having two or more chronic conditions (8, 9). In 2024, the “14th Five-Year Plan for Healthy Aging” indicated that the aging population in China will further intensify, with the proportion of people aged 60 and above exceeding 20% of the total population, and more than 78% of the older adult will have at least one chronic disease (10–12). The prevention and treatment of chronic diseases of the older adult in China is urgent.

Primary healthcare institutions serve as China’s main battleground for chronic disease prevention and control. However, some people with chronic diseases are not satisfied with the chronic disease services provided in the community (13–15). A study indicates that dissatisfaction rates among Chinese people with chronic diseases with the provision of services in community health service institutions have reached 30.34% (16). The fundamental reason for this dissatisfaction lies in the mismatch between service provision and people’s needs, leading to inefficient allocation and compromising people’s health (17, 18).

Optimizing community chronic disease management programs is pivotal in promoting primary healthcare institutions’ high-quality and effective service delivery (19, 20). A key aspect is clarifying the needs and preferences of people with chronic diseases for community health services. However, due to information asymmetry between primary healthcare institutions and people with chronic diseases, complex supply–demand relationships, and the influence of external factors, measuring the actual preferences of people with chronic diseases for community health services poses significant challenges (21, 22).

In the field of healthcare services, preference generally refers to the degree of people’ cognitive inclination toward different health conditions and healthcare services, encompassing values related to physiological, psychological, emotional, and social support aspects. Common measurement methods include the contingent valuation method, conjoint analysis, and discrete choice experiments. The contingent valuation method can only measure “preference” through trade-offs between two attributes, limiting its scope (23). Conjoint analysis requires strict mathematical derivation and proof, and it has limitations in explaining preference or choice behavior, as it does not capture the actual utility that healthcare services provide to people (24). In contrast, discrete choice experiments can comprehensively evaluate multiple attributes, face relatively weaker hypothetical biases, and closely resemble real choices. The established preferences derived from these experiments can effectively predict actual behavior and explain people’ preferences for multiple attributes, making them widely applicable in the field of healthcare services (25).

The theoretical basis of DCE originates from demand theory and McFadden’s random utility maximization theory in economics. Demand theory suggests that people base their demand for goods on specific combinations of the attributes of those goods. In contrast, utility maximization theory posits that people choose goods that maximize utility (24, 26). The advantage of DCE lies in its ability to quantitatively assess respondents’ preferences for each attribute/level under certain assumptions (25, 27). In a DCE, we ask respondents to choose between different options, each composed of attributes and levels (28–30). Their choices are then analyzed to deconstruct their preferences for the attributes and levels based on their selected service program. By designing service options with different attributes and levels, we allow chronic disease patients to choose among them, thereby studying their preferences for community health services. This approach helps identify community chronic disease management plans that better align with patient preferences from the demand side (31–33).

Our understanding of chronic disease patients’ preferences for community health services needs to be improved. Although a few studies in China have explored residents’ preferences for community health services, these studies do not specifically focus on the preferences of older adult chronic disease patients. Therefore, within the context of the Chinese healthcare system, our specific objective is to investigate the preferences of older adult patients with chronic diseases regarding community health services and to explore the heterogeneity of preferences among individuals with different types of chronic diseases. The findings of this study will provide references for optimizing chronic disease management programs in China and will inform decision-makers in developing management policies for primary healthcare. Additionally, these insights will offer valuable perspectives for regions facing similar healthcare challenges.

## 2 Materials and methods

This study applied the DCE according to the ISPOR Conjoint Analysis Good Research Practices and the WHO guidelines for using discrete choice models (34, 35). The DCE design mainly includes four stages: (1) design of attributes and levels; (2) experimental design; (3) questionnaire design and data collection; (4) analyze and interpret the data.

### 2.1 Study setting

Sichuan Province is located in Southwest China, with a population of approximately 83.68 million and a male-to-female ratio of 1.2:1. The province has a notable trend of population aging, which is largely consistent with the demographic characteristics of the entire country. The top three prevalent chronic diseases in this region are hypertension, diabetes, and coronary heart disease, reflecting, to some extent, the broader situation of chronic disease prevalence in Western China.

### 2.2 Design of attributes and levels

Identifying attributes and levels is a key step in a DCE (36–38). We conducted a systematic literature review to understand the application and development of DCE, including studies on preferences for community health services for chronic diseases. The literature review reveals that researchers commonly consider service type, medication provision, distance to healthcare facilities, and costs (39–44). Additionally, DCE attributes also need to consider China’s current policy context and demands. This study references the guidelines for primary health services from documents such as the “National Basic Public Health Service Standards (2019)” and the “China Chronic Disease Prevention and Control Plan (2017–2025),” along with the relevant policies and requirements for primary healthcare services outlined in the “14th Five-Year Plan for Healthcare Service System” issued by the Sichuan Provincial Government Office in 2022. We initially screened attributes that might be important to chronic disease patients through focused group discussions. Finally, by consulting experts in health management and policy research as well as public health specialists, we identified six attributes most likely to attract the attention of chronic disease patients: doctor type, availability of traditional Chinese medicine services, long-term prescription services, drug accessibility, appointment, referral services, and out-of-pocket costs. Table 1 shows the selected attributes and their levels.

**Table 1:** Attributes and levels of DCE.

Attributes	Levels	Definition
Type of doctor	General doctor	Types of medical registration
	Specialist	
Traditional Chinese medicine services	Obtainable	Provide traditional Chinese medicine services
	Not obtainable detail	
Long-term prescription time	One month	The effective time of a long-term prescription
	Three months	
Medicine accessibility	High	Availability of necessary chronic disease medicine/prescriptions: Low: Few medicine available for secondary and tertiary hospitals Medium: Some medicine available for secondary and tertiary hospitals High: Most medicine available for secondary and tertiary hospitals
	Medium	
	Low	
Appointment for referral services	Fast	Referral to Green Channel or appointment of specialist case
	Slow	
Cost (CNY)	40	The monthly cost of the patient
	80	
	160	

### 2.3 Experimental design

Based on the identified attributes and levels, there are 144 hypothetical scenarios (2^4^ × 3^2^ = 144). However, presenting all these scenarios to respondents is impractical. To maximize the efficiency and precision of the experimental design, we used a D-efficient design in STATA 16.0 to generate 16 choice sets (45, 46). We randomly divided the 16 choice sets into three versions to reduce the respondents’ burden. In each version, the second choice set was repeated to check the internal consistency and reliability of the questionnaire (47). Additionally, to capture the true preferences of chronic disease patients for community health services, we included an opt-out option in the experimental design (48, 49). Table 2 shows an example of a choice set.

**Table 2:** Example of choice set.

Attributes	Option ①	Option ②	Option ③
Type of doctor	General doctor	Specialist	Neither choose
Traditional Chinese medicine services	Not obtainable detail	Obtainable	
Long-term prescription time	One month	Three months	
Medicine accessibility	Low: Few medicine available for secondary and tertiary hospitals	High: Most medicine available for secondary and tertiary hospitals	
Appointment for referral services	Fast	Slow	
Cost (CNY)	80	160	
Which one do you prefer?	□	□	□

### 2.4 Questionnaire design and data collection

Recently, online questionnaires have been a convenient method for collecting DCE data (50). However, we attended face-to-face surveys because our study population is 60 and above. The survey collected demographic information (gender, age, education level, occupation, average monthly income, medical insurance, etc.) and service preference measurement (DCE questions). The study included participants who met four criteria: (1) age 60 years or older, (2) being permanent residents of the survey area, (3) having at least one chronic disease, and (4) respondents who agreed to participate in the survey.

We used the Rules of Thumb to calculate the minimum sample size required for the DCE main effects model (28, 51). The sample size depends on three factors: the levels of the DCE attributes, the number of choice sets in the DCE, and the number of options in each choice set, as follows:


$$N=\frac{500\times c}{\left(t\times a\right)}$$

Where 500 is a constant, c is the maximum number of levels in any attribute, t represents the number of DCE options in each questionnaire, and a refers to the number of options in each option. After calculation, the sample size of this study is 84. However, we increased the sample size to improve the precision of estimates for both the overall sample and subgroups.

A multistage stratified random sampling method was used to determine the sample. First, two cities in Sichuan Province were randomly selected, followed by two districts within each city. Next, two to three communities were randomly selected within each district. In total, we identified 8 urban sample sites and 2 rural sample sites, with approximately 50 respondents in each site. To increase the response rate and ensure data quality, all surveyors received standardized training before the formal survey commenced, and only those who passed the assessment were allowed to join the survey team. During the survey, the surveyors were required to strictly follow the survey protocol, with no arbitrary changes to survey locations or subjects. After completing each community survey, reviewers were responsible for consolidating the results and standardizing the indicators and written formats. Community officials assisted surveyors in communicating with participants who had strong local accents during the DCE survey. Data collection occurred from March to May 2024. A total of 500 questionnaires were distributed, with 456 returned, achieving an effective response rate of 91.2%. A double-entry method was employed for data entry to ensure the accuracy of both the field survey and data recording, with discrepancies resolved by a third researcher.

### 2.5 Analyze and interpret the data

We used Stata 16.0 statistical software for data analysis. The mixed logit model, which allows parameters to vary randomly among individuals, is commonly used in DCE studies because it captures individual heterogeneity through the distribution of model parameters (mean and standard deviation). We applied the mixed logit model to estimate the respondents’ preference levels for each attribute. We calculated each attribute’s relative importance (RI) by dividing the utility difference between its lowest and highest levels by the total utility difference across all attributes (52, 53). Additionally, we calculated the willingness to pay (WTP) by determining the marginal substitution rate between non-monetary attributes and monthly out-of-pocket costs (54).

### 2.6 Mixed logit model

We set the monthly out-of-pocket cost as a fixed parameter, and all other attributes as random parameters. The specific expression is:


$$U_{ijt}=V_{ijt}\left(X_{ijt},\beta \right)+\varepsilon_{ijt};i=1,\cdots ,395;j=1,2,3;t=1,\cdots ,6$$

Where $V_{ijt}$ indicates the fixed term of $U_{ijt}$ , $\varepsilon_{ijt}$ is the error term, $X_{ijt}$ is a vector of variables representing the attributes of alternative *j* and β is a vector of coefficients. The mean value and standard deviation appeared in the model estimation results.

### 2.7 WTP

WTP refers to the willingness of people with chronic diseases to pay for acquiring or improving a health service. The expression is:


$${WTP}_{Xa}=\beta_{Xa}÷\beta_{\mathrm{cost}}$$

$\beta_{Xa}$ is the regression coefficient of the other attribute, and $\beta_{\mathrm{cost}}$ is the regression coefficient of the monthly out-of-pocket cost. The ratio of the regression coefficient of a particular attribute to the monthly out-of-pocket cost represents the WTP of people with chronic diseases to obtain or improve that attribute.

## 3 Results

### 3.1 Basic characteristics of survey participants

A total of 456 participants who provided informed consent were eligible and completed the survey, with 61 excluded due to failure to pass the rationality test. Finally, data from 395 people with chronic diseases were analyzed. Table 3 shows the characteristics of the participants.

**Table 3:** Basic characteristics of survey objects.

Characteristics	Full sample: *n* = 456	Analysis sample: *n* = 395	Excluded sample: *n* = 61
Sex			
Male	195 (42.8)	168 (42.5)	27 (44.3)
Female	261 (57.2)	227 (57.5)	34 (55.7)
Age			
60–70	307 (67.3)	267 (67.6)	40 (65.6)
Above 70	149 (32.7)	128 (32.4)	21 (34.4)
Educational level			
Junior middle or below	170 (37.3)	157 (39.7)	13 (21.3)
Senior high school or technical secondary school	223 (48.9)	178 (52.7)	45 (73.8)
University and above	63 (13.8)	60 (7.6)	3 (4.9)
Occupation			
Employed	100 (21.9)	93 (23.5)	7 (11.5)
Retire	356 (78.1)	302 (76.5)	54 (88.5)
Marital status			
Married	329 (72.1)	306 (77.5)	23 (37.7)
Other	127 (27.9)	89 (22.5)	38 (62.3)
Living condition			
Live alone	105 (23.0)	78 (19.7)	27 (44.3)
Live with others	351 (77.0)	317 (80.3)	34 (55.7)
Income per month CNY			
<2,000	219 (48.0)	197 (49.9)	22 (36.1)
2,000–4,000	169 (37.1)	142 (35.9)	27 (44.3)
>4,000	68 (14.9)	56 (14.2)	12 (19.7)
Health insurance Type			
Employee insurance	174 (38.1)	153 (38.7)	21 (34.4)
Resident insurance	195 (42.8)	164 (41.6)	31 (50.8)
Others	87 (19.1)	78 (19.7)	9 (14.8)
Chronic disease Type			
Type I	258 (56.6)	236 (59.7)	22 (36.1)
Type II	127 (27.9)	114 (28.9)	13 (21.3)
Type III	108 (23.7)	90 (22.8)	18 (29.5)

CNY, Chinese yuan; Type I, type 2 diabetes mellitus combined with hypertension; Type II, hypertension combined with coronary heart disease; Type III, type 2 diabetes mellitus combined with hypertension and coronary heart disease.

### 3.2 Mixed logit model estimation

In the mixed logit model, the type of doctor, provision of traditional Chinese medicine services, extended prescription services, medicine accessibility, appointment referral services, and monthly out-of-pocket significantly influenced chronic disease patients’ preferences for community healthcare services (Table 4). The degree of influence on respondents’ preferences is related to the size and direction of the regression coefficient. A positive direction indicates a positive preference for the attribute, while a negative direction indicates a negative preference. A larger absolute value of the regression coefficient indicates a greater impact of the attribute on respondents’ utility, and vice versa. As shown in Table 4, people with chronic diseases exhibited a stronger preference for general doctor (coefficient = 0.351, *p* = 0.012), traditional Chinese medicine services (coefficient = 1.465, *p* < 0.001), extended prescription times (coefficient = 0.682, *p* < 0.001), high medicine accessibility (coefficient = 2.761, *p* < 0.001), and appointment referral services (coefficient = 2.385, *p* < 0.001). In terms of monthly out-of-pocket, respondents preferred healthcare services with lower costs (coefficient = −0.037, *p* < 0.001). Table 4 also shows the willingness of chronic patients to pay for each attribute. People with chronic diseases are most willing to pay for high medicine accessibility (WTP = 74.04). Next was the appointment referral service (WTP = 63.94) and traditional Chinese medicine services (WTP = 39.27).

**Table 4:** Regression results of overall choice preference of people with chronic diseases for community health services.

Attributes levels	Coefficient	SD	*p*-value	WTP
Cost	−0.037	0.002	<0.001	–
Type of doctor (ref: specialist)				
General doctor	0.351	0.140	0.012	9.41
Traditional Chinese medicine services (ref: not obtainable detail)				
Obtainable	1.465	0.148	<0.001	39.27
Long-term prescription (ref: one month)				
Three months	0.682	0.130	<0.001	18.28
Medicine accessibility (ref: low)				
Medium	1.756	0.155	<0.001	47.09
High	2.761	0.216	<0.001	74.04
Appointment for referral services (ref: slow)				
Fast	2.385	0.171	<0.001	63.94
Alternative Specific Constant	−1.080	0.147	<0.001	–
Model specification				
Log likelihood	−1302.844			
Akaike information criterion	2633.687			
Bayesian information criterion	2728.203			

### 3.3 Relative importance of attributes

**Figure 1:**
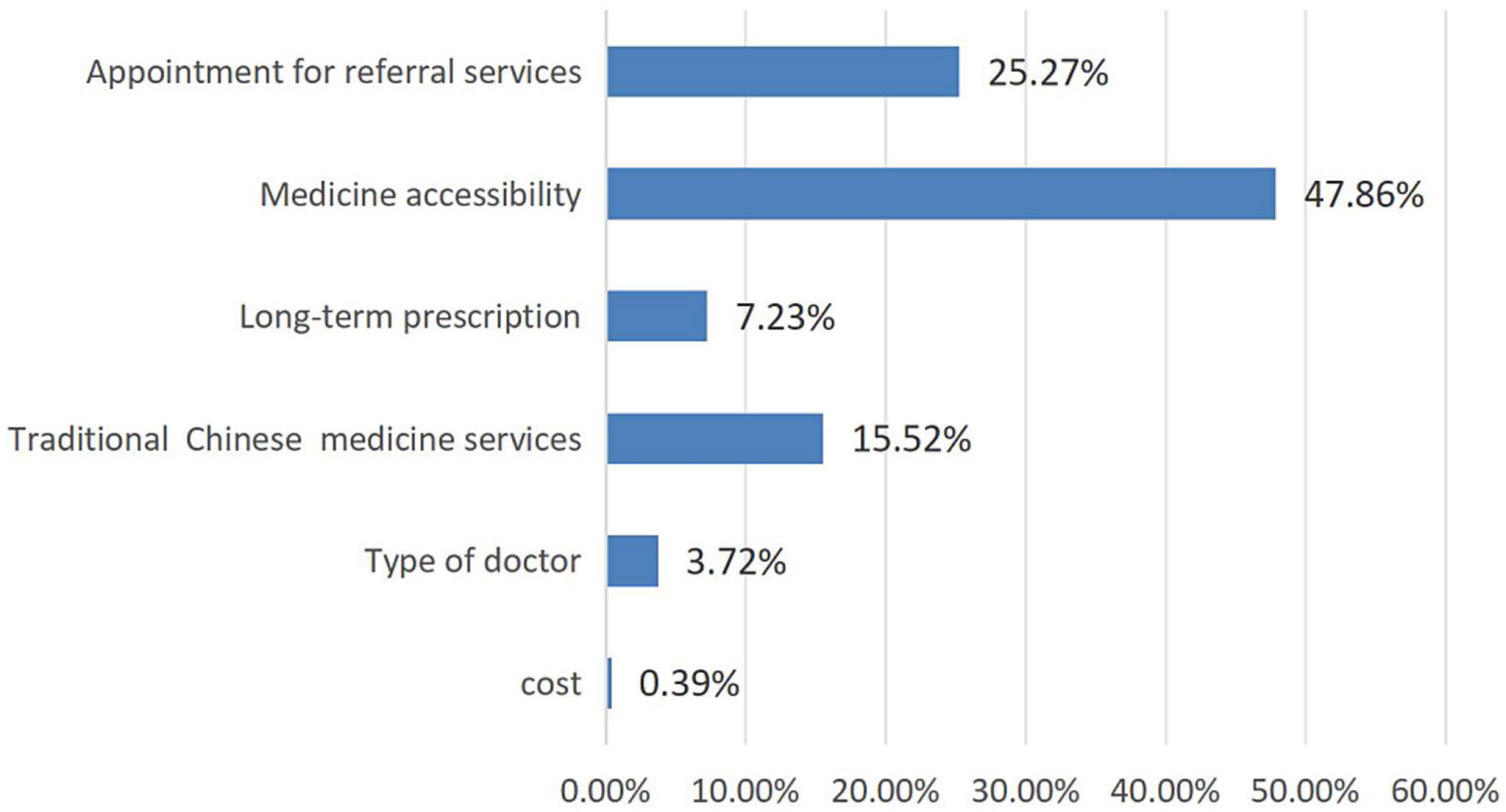
Relative importance of attributes.

Calculating of the relative importance of attributes involves dividing the range of each attribute by the sum of the ranges of all attributes. Figure 1 illustrates the relative importance of the attributes among respondents, showing that they value medicine accessibility the most (47.86%), followed by appointment for referral services (25.27%) and traditional Chinese medicine services (15.52%).

### 3.4 Subgroup analysis

**Figure 2:**
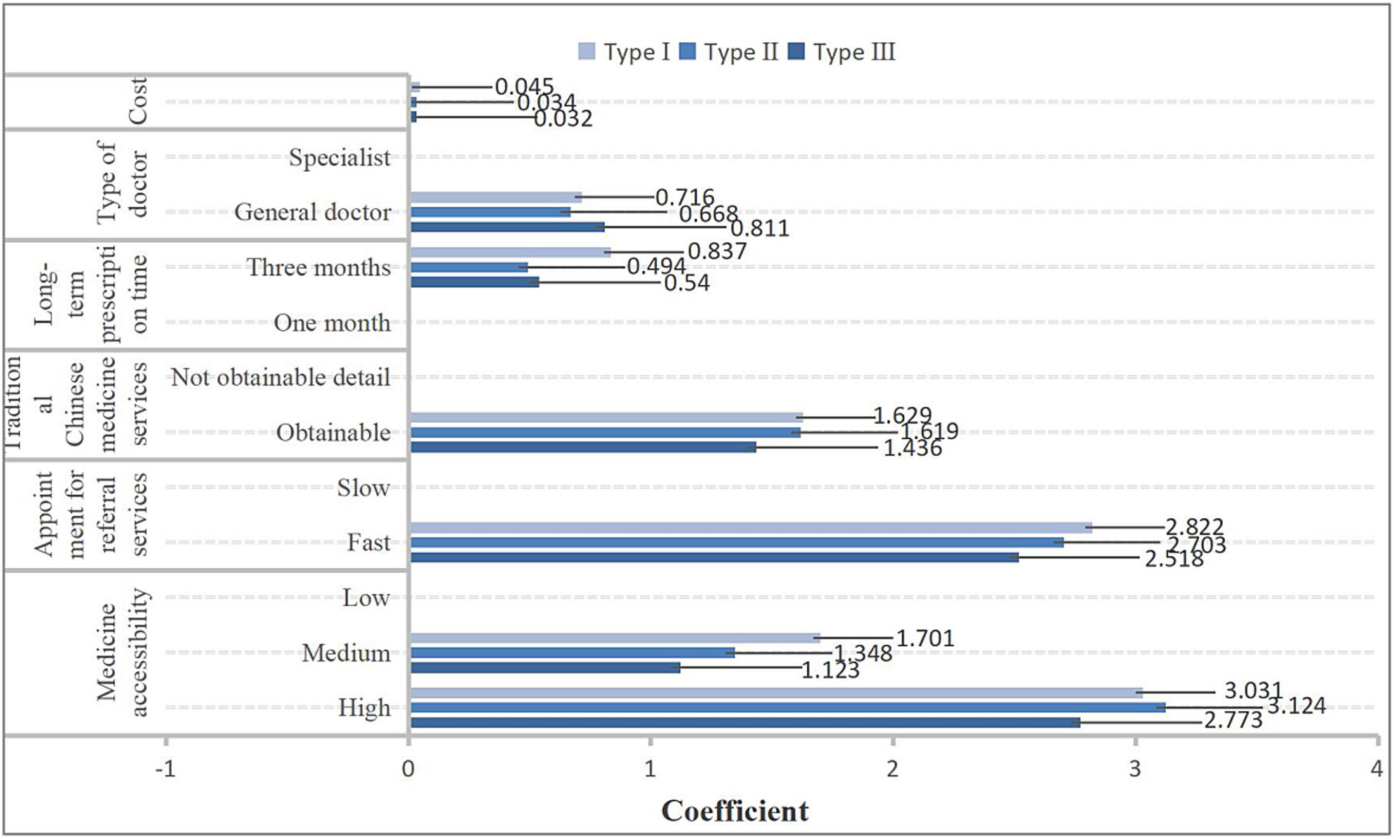
The results of preference for community health services among different types of people with chronic comorbidities.

**Figure 3:**
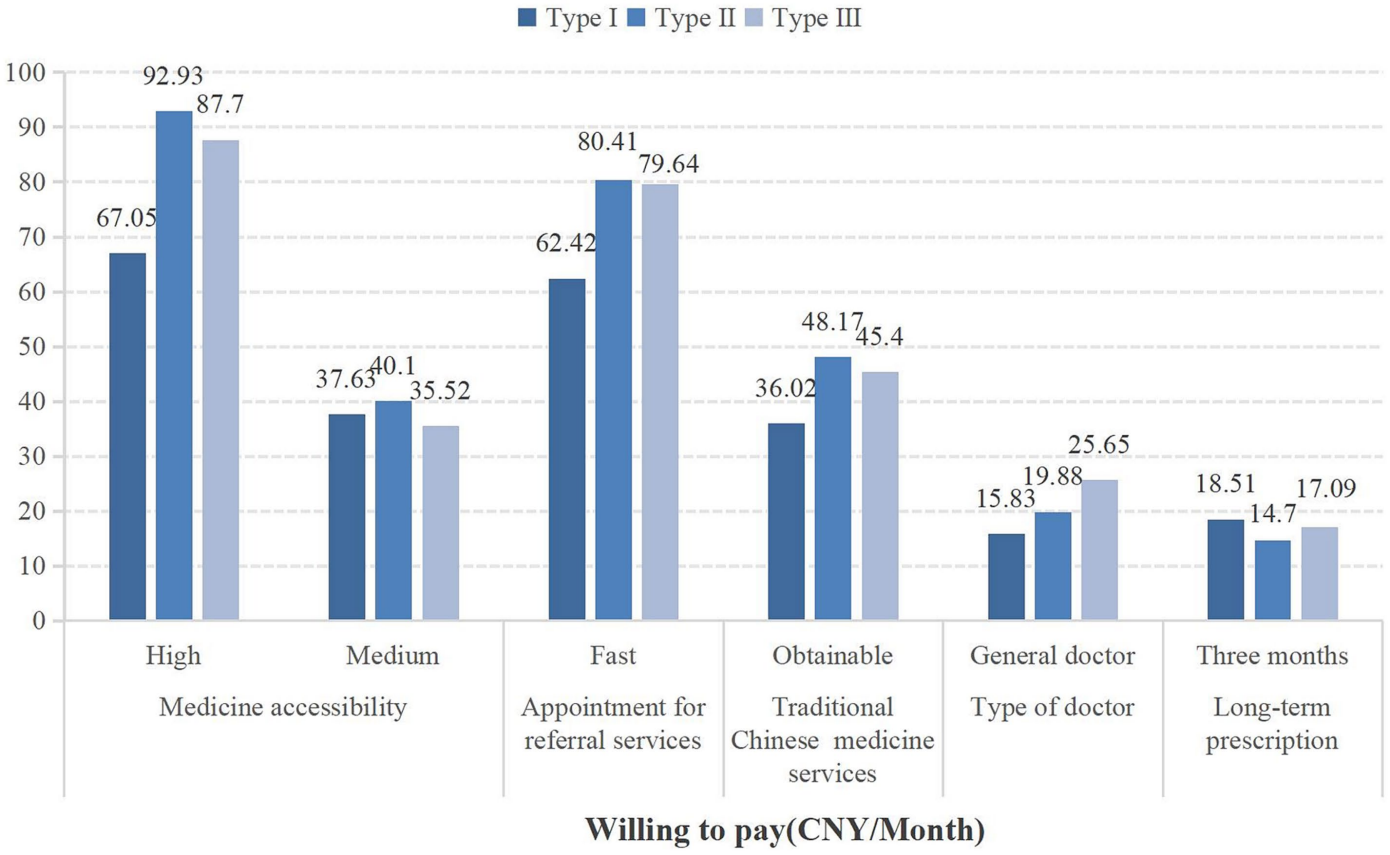
The willingness to pay for community health services among different types of people with chronic comorbidities.

Chronic diseases such as type 2 diabetes combined with hypertension, hypertension combined with coronary heart disease, and type 2 diabetes combined with hypertension and coronary heart disease are categorized into type I, type II, and type III populations, respectively. Type I people, type II people, and type III people all prioritize services with high medicine accessibility, as shown in Figure 2. At the same time, patients with different types of comorbid chronic diseases have the highest willingness to pay for drug accessibility (Figure 3).

## 4 Discussion

Our study utilized DCE to elicit the preferences of older adult chronic disease patients in China for community health services. Through literature review and expert consultations, we identified six attributes most likely to influence respondents’ preferences: doctor type, traditional Chinese medicine services, long-term prescription services, medicine accessibility, appointment and referral services, and monthly out-of-pocket costs. The survey revealed an interesting phenomenon: medicine accessibility emerged as the most critical attribute for respondents, followed by appointment and referral services and traditional Chinese medicine services. Doctor type and costs had some influence but were not the most significant factors. Secondly, we explored the willingness to pay of respondents for these attributes. The highest WTP was observed for the accessibility of medications, followed by referral appointment services and traditional Chinese medicine services, while the lowest willingness to pay was for the type of physician. These results reflect the preferences of chronic disease patients in Western China regarding community health services, providing insights for decision-makers to optimize chronic disease management in Chinese communities. Furthermore, this research can serve as a reference for preference studies in other regions facing similar healthcare challenges.

Due to the limitations of the model itself, we discussed respondents’ preferences for community health services in hypothetical scenarios, which may differ to some extent from real-world situations. However, through a preliminary literature review and expert consultations, we gained an understanding of the current needs of chronic disease patients for community health services. Additionally, we took the current policy context into account and included an opt-out option, providing respondents with sufficient freedom of choice, thus closely approximating real-life situations. By focusing on measuring respondents’ preferences for community health services and estimating the relative importance of different attributes, these constraints do not affect our main conclusions (55).

Undoubtedly, medicine accessibility was the most crucial factor for respondents. Interestingly, regardless of the type of chronic disease, respondents consistently preferred services with high medicine accessibility and were willing to pay more for such services. For example, as shown in Table 3, respondents valued services with high medicine accessibility the most (coefficient = 2.761, *p* < 0.001) and had a willingness to pay 74.04 CNY for these services. These findings echo previous studies (56–59). For example, a prospective epidemiological study of essential drugs for chronic diseases showed that primary healthcare institutions with a greater variety and more comprehensive supply of essential medicine are more likely to be preferred by people (60). Chinese scholar Tian also found that primary healthcare institutions with a broader and more complete range of essential medicines were more favored by residents (61). These results suggest that adequate drug availability can enhance respondents’ willingness to seek community-based care and promote better chronic disease management in the community (62, 63). Therefore, policymakers could consider increasing funding for primary health service programs and expanding the coverage of essential medications in primary healthcare institutions to meet the medication needs of chronic disease patients.

Secondly, respondents exhibited a strong preference for appointment and referral services. This preference may stem from efficient appointment and referral services providing a better patient experience by saving time (43). As shown in Table 3, respondents preferred quick appointment and referral services (coefficient = 2.385, *p* < 0.001) and had a high willingness to pay (WTP = 63.94 CNY) for appointment and referral services. This result indicates that respondents recognize the importance of appointment and referral systems in community health services, believing such systems can better serve them. One study supports our findings, suggesting that smooth referral pathways between primary healthcare institutions and higher-level hospitals can positively promote community chronic disease management (64). Additionally, residents with poorer health status demand more referrals and other community health services (65). This result demonstrates that improving the efficiency of appointment and referral services can assist in the community management of older adult chronic disease patients (66, 67). Policymakers should improve the referral appointment system, streamline the process of bidirectional referrals, and establish an online information platform for referral appointments to facilitate the referral process and enhance efficiency.

Traditional Chinese medicine services are also highly valued by older adult chronic disease patients. Our study found that these patients prefer community health services offering Traditional Chinese medicine, indicating trust in Traditional Chinese medicine and an increasing emphasis on its role in treating and preventing chronic diseases. This is a noteworthy finding. With changes in the medical model, integrated traditional Chinese and Western medical services play an increasingly important role in prevention, treatment, and rehabilitation (68, 69). Scholar Zhang also found that residents had a highly preferred for integrated Chinese and Western medical services, believing that such services could better serve their needs (70).

However, only traditional Chinese medicine hospitals in China currently have comprehensive traditional Chinese medicine management capabilities (69). Community healthcare institutions need more traditional Chinese medicine service facilities and more efficient management, making it challenging to meet the demand for traditional Chinese medicine services among people with chronic diseases. Instead of forming a unique advantage in traditional Chinese medicine service provision, this situation has hindered the management of chronic diseases in the community (71, 72). Our study results indicate that the government should provide policy guidance to community health service institutions regarding the provision of traditional Chinese medicine services. Strengthening the application of traditional Chinese medicine services in community chronic disease management can contribute to optimizing community chronic disease management (73, 74). For instance, establishing traditional Chinese medicine service centers to standardize traditional Chinese medicine management, or building an information platform for traditional Chinese medicine services to achieve information sharing and connectivity, providing high-quality and effective traditional Chinese medicine services to more chronic disease patients.

Our subgroup analysis revealed that respondents with different types of chronic diseases have varying preferences for community health services. All three types of respondents preferred high medicine accessibility the most, followed by appointment, referral services, and traditional Chinese medicine services. However, there was heterogeneity in preferences for doctor type and long-term prescription services. Type I respondents were more inclined to choose long-term prescription services, while Type II and Type III respondents preferred doctor type. This may be related to the nature of chronic diseases (41). Diabetes and hypertension require long-term medication, so individuals with these conditions might place more importance on the duration of prescriptions. Moreover, there were differences in willingness to pay (WTP) for community health services among the three types of respondents. Type II respondents had a higher WTP for services with high medicine accessibility (92.93 CNY) compared to Type I (67.05 CNY) and Type III (87.70 CNY) respondents. This variation may be due to differences in preference weights and economic status (75).

Additionally, the type of physician, extended prescription services, and patient out-of-pocket expenses all influence the preference for community health services among people with chronic diseases. Compared to specialist physicians, people with diabetes and hypertension comorbidity are willing to pay 15.83 CNY for general practitioners. Relative to a one-month extended prescription service, people with diabetes and hypertension comorbidity demonstrate a WTP of 18.51 CNY for a three-month extended prescription service. Additionally, regardless of the type of chronic disease, people with chronic diseases generally prefer lower-cost community health services. It indicates that focusing on meeting people’s needs can effectively promote the prevention and management of chronic diseases in communities (76). From the perspective of preference weights and WTP, physician type, extended prescription services, and patient out-of-pocket expenses on people with chronic diseases may not be as significant as the impact of attributes such as drug accessibility and referral appointments. However, multiple incentives can be implemented together (77, 78). For example, providing services with high drug accessibility while simultaneously offering general practitioner services can better promote community management of people with chronic comorbidities.

This study has certain limitations. Firstly, we conducted face-to-face interviews, which might have introduced potential biases due to self-reported data. Secondly, our research mainly focused on primary healthcare in Southwest China. Although these findings may have implications for other regions of China, further studies in other cities are needed. Thirdly, Due to sample size limitations, the proportion of rural residents in our sample is relatively small, making it impossible to compare the preferences of urban and rural residents. There may be differences in preferences between these two groups. In the future, we plan to expand our survey of rural residents to further explore the heterogeneity in preferences between urban and rural populations. Meanwhile, we did not account for socioeconomic factors, such as education level and economic stability, which may also influence the preferences of chronic disease patients. Additionally, the discrete choice experiment was conducted in hypothetical scenarios, so respondents’ actual preferences and willingness to pay might differ from their real-world decisions. Lastly, we plan to further investigate the impact of socioeconomic factors on the preferences of older adult patients with chronic diseases. If conditions permit, we will expand our sample size and conduct similar studies in other regions of China to further explore the applicability of our findings.

## 5 Conclusion

In summary, addressing the needs of people with chronic diseases and driving community management based on these needs is the current focus of chronic disease prevention and management. By accurately identifying demand, improving referral appointment systems, accelerating the integration of traditional Chinese medicine into community-based chronic disease prevention and management, and implementing various incentive policies, people with chronic diseases can be guided to seek medical treatment in an orderly and efficient manner, leading to the rational utilization of medical resources and promoting the implementation of tiered diagnosis and treatment in China. These findings will provide valuable insights for policymakers to optimize the current management of chronic diseases in Chinese communities.

## Data Availability

The original contributions presented in the study are included in the article/supplementary material, further inquiries can be directed to the corresponding author.
